# STROKE PREVENTION EDUCATION IN AFRICAN AMERICANS: A COMMUNITY-BASED PARTICIPATORY FEASIBILITY STUDY

**DOI:** 10.1093/geroni/igae098.0259

**Published:** 2024-12-31

**Authors:** Niloufar Hadidi, Clarence Jones, Zachary Taylor, Emily Gorzycki, Allison Pasdo, Olga Gurvich, Susan Everson-Rose

**Affiliations:** University of Minnesota, Minneapolis, Minnesota, United States; Hue-MAN Partnership, Minneapolis, Minnesota, United States; Allina Health, Minneapolis, Minnesota, United States; Abbott Northwestern Hospital, Minneapolis, Minnesota, United States; University of Minnesota, Minneapolis, Minnesota, United States; University of Minnesota, Minneapolis, Minnesota, United States; University of Minnesota Medical School, Minneapolis, Minnesota, United States

## Abstract

**Research aim:**

To assess the feasibility and acceptability of a Stroke Champion “train-the-trainer” program to support implementation of stroke prevention education among African Americans (AA) residing in the Minneapolis-St. Paul metropolitan area. Significance: AAs are fifty percent more likely to experience stroke than Whites. This disparity is partially attributed to higher prevalence of modifiable stroke risk factors such as hypertension, diabetes, and obesity among AA communities. Training community members to disseminate health information is an evidence-based, community-specific health promotion strategy. Stroke Champions - individuals from the community with a desire to help improve the health of their community – could be trained and empowered to deliver stroke recognition and prevention education to peers.

**Methods:**

Twelve AA Stroke Champions were recruited to assess the feasibility of educating community on 1) stroke warning signs and risk factors; and 2) strategies to mitigate stroke risk factors. A one arm pre-post-test design was used. Findings: We successfully developed and implemented a stroke education website for community education. Stroke Champions reported the website was helpful, easy to use, and a valuable health information resource They stated that they needed more support from the research team to deliver the education to peers in the community.

**Conclusion:**

Engaging Stroke Champions is potentially beneficial in increasing stroke prevention knowledge. However, Stroke Champions need more knowledge and facilitation support from the research team to effectively engage and educate peers. Implication: Community engagement is an important way to empower individuals to take charge of their health and reduce stroke burden.

